# The predictive value of relative wall thickness on the prognosis of the patients with ST-segment elevation myocardial infarction

**DOI:** 10.1186/s12872-023-03379-5

**Published:** 2023-07-31

**Authors:** Ying Zhang, Shuaihua Qiao, Han Hao, Qiaoling Li, Xue Bao, Kun Wang, Rong Gu, Guannan Li, Lina Kang, Han Wu, Zhonghai Wei

**Affiliations:** Department of Cardiology, Nanjing Drum Tower Hospital, The Affiliated Hospital of Nanjing University Medical School, Nanjing, 210008 China

**Keywords:** Relative wall thickness, ST-segment elevation myocardial infarction, Primary percutaneous coronary intervention, Prognosis, Echocardiography

## Abstract

**Objective:**

The study aimed to evaluate the prognostic value of relative wall thickness (RWT) in the patients with ST-segment elevation myocardial infarction (STEMI).

**Methods:**

A total of 866 patients with STEMI admitted in Nanjing Drum Tower Hospital, The Affiliated Hospital of Nanjing University Medical School from November 2010 to December 2018 were enrolled in the current study retrospectively. Three methods were used to calculate RWT: RWT_PW_, RWT_IVS+PW_ and RWT_IVS_. The included patients were divided according to the median values of RWT_PW_, RWT_IVS+PW_, and RWT_IVS_, respectively. Survival analysis were performed with Kaplan–Meier plot and multivariate Cox proportional hazard model was established to evaluate the adjusted hazard ratio of the three kinds of RWT for all cause death, cardiac death and MACE (major adverse cardiac death).

**Results:**

There was no significance for the survival analysis between the low and high groups of RWT_PW_, RWT_IVS+PW_ and RWT_IVS_ at 30 days and 12 months. Nonetheless, the cumulative incidence of all cause death and cardiac death in the low group of RWT_PW_ and RWT_IVS+PW_ was higher than those in the high group at 60 months. The cumulative incidence of MACE in the low group of RWT_PW_ was higher than the high group at 60 months. Multivariate Cox regression model showed that RWT_PW_ were inversely associated with long-term cardiac death and MACE in STEMI patients. In the subgroup analysis, three calculations of RWT had no predictive value for the patients with anterior myocardial infarction. By contrast, RWT_PW_ was the most stable independent predictor for the long-term outcomes of the patients with non-anterior myocardial infarction.

**Conclusion:**

RWT_PW_, RWT_IVS+PW_ and RWT_IVS_ had no predictive value for the long-term clinical outcomes of patients with anterior myocardial infarction, whereas RWT_PW_ was a reliable predictor for all cause death, cardiac death and MACE in patients with non-anterior myocardial infarction.

## Key message

RWT is an index which can quantified the concentricity or eccentricity of the left ventricular using a simple formula.

There are three methods to calculate RWT: RWT_PW_ = 2 × PWth/LVDd; RWT_IVS+PW_ = (IVSth + PWth)/LVDd; RWT_IVS_ = 2 × IVSth/LVDd(IVSth: intraventricular septal thickness; LVDd: LV diameter at the end of diastole; PWth: posterior wall thickness).

From our study we concluded that RWT_PW_, RWT_IVS+PW_ and RWT_IVS_ had no predictive value for the long-term clinical outcomes of patients with anterior myocardial infarction, whereas RWT_PW_ was the most effective predictor of patients with non-anterior myocardial infarction.

## Introduction

The primary percutaneous coronary intervention (pPCI) has been the first line therapy for ST-segment elevation myocardial infarction (STEMI) for decades, which has remarkably reduced the in-hospital mortality of the patients with STEMI. However, the patients still face an elevated risk of subsequent cardiovascular events [1, 2]. The risk stratification of these patients remains a challenge and is important to the subsequent treatment and health management [3]. After acute myocardial infarction (MI), ventricular remodeling occurs promptly, such as change of the structure, morphology and ventricular function, which is a manifestation of left ventricular enlargement, decreased left ventricle ejection fraction (LVEF) and abnormal regional wall motion [4]. Previous studies have identified various predictors for the clinical outcomes of STEMI, including LVEF, brain natriuretic peptide (BNP), estimated glomerular filtration rate (eGFR) and high-sensitive C-reactive protein [5–8]. However, these indices are unable to reflect the pattern of the ventricular remodeling. Relative wall thickness (RWT) is an index which can quantify the concentricity or eccentricity of the left ventricular using a simple formula. There are three methods to calculate RWT: RWT_PW_ = 2 × PWth/LVDd; RWT_IVS+PW_ = (IVSth + PWth)/LVDd; RWT_IVS_ = 2 × IVSth/LVDd (IVSth: intraventricular septal thickness; LVDd: LV diameter at the end of diastole; PWth: posterior wall thickness; PW refers to LVPW) [9]. Previous studies have found that a higher RWT was associated with a poorer prognosis of patients with acute decompensated heart failure (including heart failure with preserved or reduced ejection fraction). Besides, a lower RWT was also related to a higher incidence of ventricular arrhythmia in patients with left ventricular dysfunction [9, 10]. So far, RWT_PW_ is the most widely used method in the clinical practice. Nevertheless, the predictive value of RWT as calculated by different methods has not been reported for the clinical outcomes of the patients with STEMI. Thus, we carried out the current study to evaluate the prognostic value of RWT in a cohort of STEMI patients.

## Methods

### Study population

The diagnosis of STEMI was based on the criteria of American College of Cardiology/American Heart Association (ACC/AHA) and the European Society of Cardiology (ESC) [3, 11]. This is a single-center observational study. The data of the study population were obtained from the databases in our institution. The ethics has been approved by the Medical Ethics Committee of Nanjing Drum Tower Hospital, Medical School of Nanjing University (2019–190-01). The relevant data were published with the verbal consent by the participants and has been approved by the ethics committee.

The including criteria were as follows: (1) patients between 18 and 90 years; (2) all patients presented acute chest pain in the emergency department of our hospital; (3) STEMI was diagnosed by electrocardiography (ECG) in emergency department; (4) the patients were eligible for pPCI and willing to accept the procedure.

The exclusion criteria were as follows: (1) the patients did not undergo the emergency angiography; (2) the patients did not undergo the emergency revascularization after angiography; (3) the patients were suitable for the emergency coronary artery bypass graft surgery (CABG); (4) the patients were lost to follow-up [12].

**Fig. 1:**
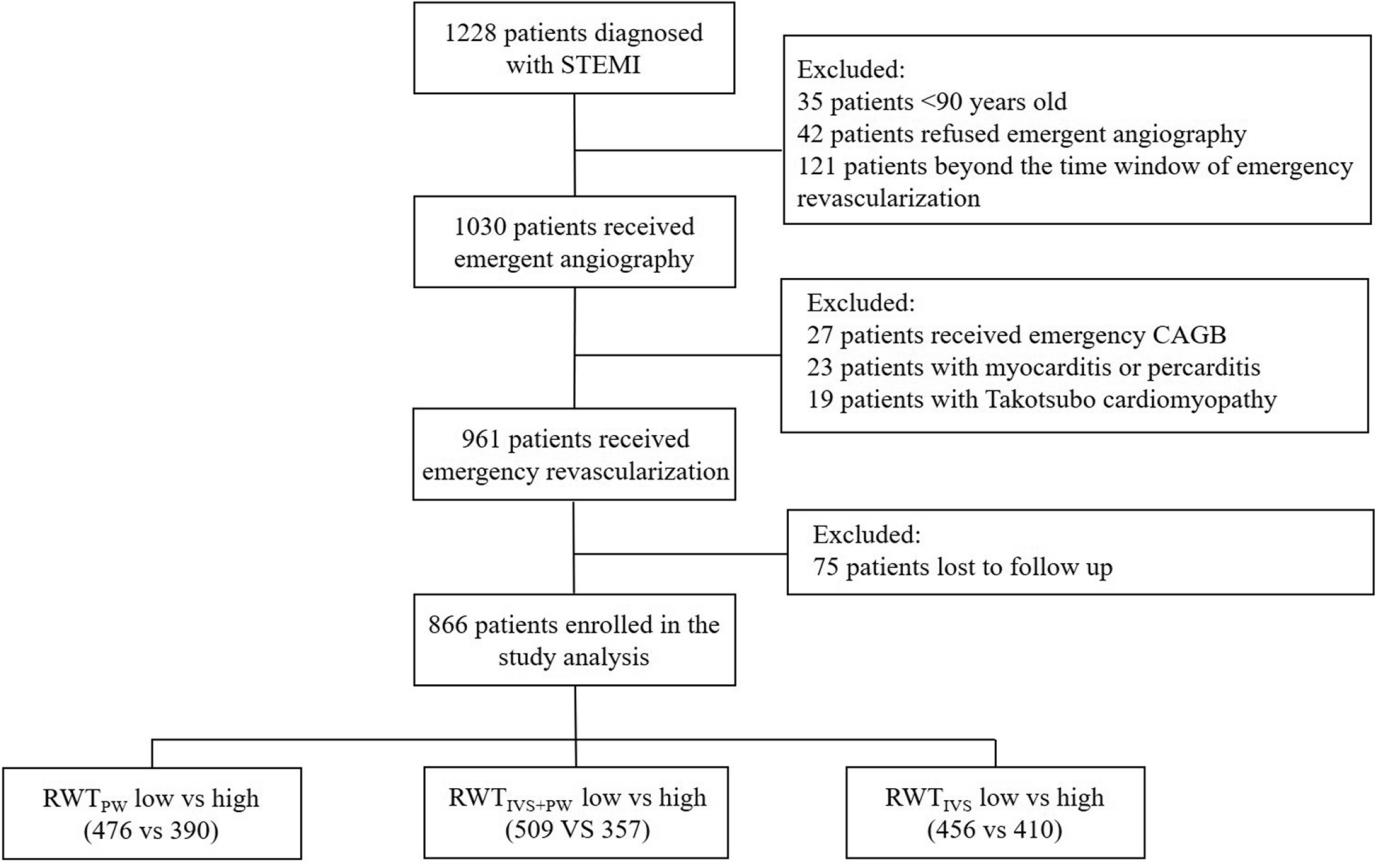
Flowchart of patients inclusion. The data was divided into ‘low’ and ‘high’ according to the median. STEMI: ST-segment elevation myocardial infraction; CABG: coronary artery bypass grafting; RWT: relative wall thickness

Consequently, 866 patients with STEMI admitted in Nanjing Drum Tower Hospital, The Affiliated Hospital of Nanjing University Medical School from November 2010 to January 2018 were enrolled in the current study analysis. The included patients were divided according to the median values of RWT_PW_, RWT_IVS+PW_, and RWT_IVS_, respectively. The enrollment flow chart was shown in the Fig. 1.

### Study protocol

ECG was performed within 10 min for all patients with acute chest pain. STEMI was defined as ST segment elevation at the J point in at least 2 contiguous leads of above 2 mm in men or above1.5 mm in women at V2 and V3 lead and/or of above 1 mm in other leads. The new onset of left bundle branch block on the ECG was considered as STEMI [13]. The patients were taken to catheterization laboratory immediately after taking 300 mg aspirin and 180 mg ticagrelor/600 mg clopidogrel. Revascularization strategy was individualized according to the angiography results and interventionists’ decisions. Standardized treatments of STEMI during and after hospitalization were in accordance to the guidelines. All patients received cardiac function assessment within 48 h after admission. Philips IE33 ultrasound machine was used for echocardiography examination and Simpson algorithm was used to identify the left ventricular ejection fraction. All the procedures were accomplished by experienced and qualified doctors.

### Follow up

The study population was followed up via telephone or outpatient department. The follow-up was carried out until 1^st^ March, 2022. Endpoints include all cause death, cardiac death, and major adverse cardiac events (MACE) at 30 days, 12 months and 60 months. All cause death was defined as death to any causes. Cardiac death was defined as the death due to any cardiac diseases, such as myocardial infarction, cardiac rupture, arrhythmia, heart failure and so on. MACE was defined as a composite of cardiac death, recurrent angina or MI, exacerbation of heart failure and non-fatal ischemic stroke.

### Statistical analysis

The continuous variables were presented as the mean ± standard deviations (SD) or median and interquartile range (IQR) according to the data distribution. The categorical variables were presented as frequency and percentages. In two-group comparisons, Student’s t-test and the Mann–Whitney U test were used to compare normally distributed and non-normally distributed continuous variables, respectively. χ^2^ test or Fisher’s exact test was used for categorical variables. Survival analysis was performed by Kaplan–Meier plot and Log rank test. Cox proportional hazard models were established to estimate the adjusted hazard ratio (HR) of RWT for different endpoints. The restricted cubic spline models with 3 knots placed at 10^th^, 50^th^ and 90^th^ percentile of RWT were used to evaluate the association between RWT (as a continuous term) and the endpoints. There were total 25 variables including 14 continuous variables (age, systolic bleed pressure, heart rate, shock index, creatinine, LDL-C, LVEF, LVDd, IVSth, PWth, LA, RWT_PW_, RWT_IVS+PW_ and RWT_IVS_) and 11 categorical variables (male sex, shock, hypertension, diabetes, hyperlipidemia, prior stroke, smoking, family history, anterior wall, multivessel lesions, Killip classification). After univariate analysis, the covariates with *P* < 0.1 and the covariates with *P* > 0.1 but with clinical significance were extracted for multivariate regression analysis. An interaction analysis model was established to study whether there is interaction between variables. A two-tailed *P* value < 0.05 is considered as statistically significant. The statistical analysis was performed by SPSS 25.0 (SPSS Inc., Chicago, Ill., USA) and R 4.0 (R core team 2020, R Foundation for statistical computing, Vienna, Austria).

## Results

### Basic characteristics of study cohort

The median age of the patients was 65 years (IOR: 54-74 years) and 80.3% were male. The median value of the RWT_PW_, RWT_IVS+PW_ and RWT_IVS_ for the three different calculation methods was 0.32, 0.33 and 0.33, respectively. According to the three median values, the patients were divided into low group and high group, respectively. No matter which calculation method was used, the patients in the low group had lower LVEF, IVSth, PWth and higher LVDd value than those in the high group. In addition, less patients had hypertension and more patients had anterior myocardial infarction in the low group as compared to the high group. The patients in low group of RWT_PW_ had lower systolic blood pressure, higher value of left atrium (LA), low density lipoprotein cholesterol (LDL-C) and shock index than high group. The patients in low group of RWT_IVS+PW_ had less patients with diabetes than high group. The patients in low group of RWT_IVS_ had less patients with diabetes, higher LA value and older than high group (Table 1).

**Table 1:** Comparison of baseline characteristics of patient cohort in the three calculation methods

	RWT_PW_			RWT_IVS+PW_			RWT_IVS_		
	Low Group (≤ 0.32,*n* = 476)	High Group (> 0.32,*n* = 390)	*P* value	Low Group (≤ 0.33,*n* = 509)	High Group (> 0.33,*n* = 357)	*P* Value	Low Group (≤ 0.33,*n* = 456)	High Group (> 0.33,*n* = 410)	*P* Value
Age(years)	64.71(55–74)	63.1(53–74)	0.114	64.3(54–74)	63.37(54–73)	0.285	64.71(54–75)	63.07(53.5–72.5)	**0.039**
Male sex,n(%)	353(79.9)	337(81.0)	0.673	372(80.0)	318(80.9)	0.736	337(79.5)	353(81.3)	0.494
Shock,n(%)	29(6.6)	18(4.3)	0.151	29(6.2)	18(4.6)	0.288	27(6.4)	20(4.6)	0.257
Bleeding,n(%)	15(3.7)	7(1.8)	0.089	11(2.6)	11(2.9)	0.790	11(2.8)	11(2.6)	0.873
History of cancer,n(%)	11(2.7)	4(1.0)	0.073	10(2.3)	5(1.3)	0.281	9(2.3)	6(1.4)	0.361
Hypertension,n(%)	254(57.5)	274(65.9)	**0.011**	263(56.6)	265(67.4)	**0.001**	235(55.4)	293(67.5)	** < 0.001**
Diabetes,n(%)	116(26.2)	111(26.7)	0.884	101(21.7)	126(32.1)	**0.001**	92(21.7)	135(31.1)	**0.002**
Hyperlipidemia,n(%)	64(14.5)	69(16.6)	0.394	67(14.4)	66(16.8)	0.336	59(13.9)	74(17.1)	0.205
Prior stroke,n(%)	60(13.6)	53(12.7)	0.718	64(13.8)	49(12.5)	0.576	56(13.2)	57(13.1)	0.974
Smoking,n(%)	256(57.9)	226(54.3)	0.289	272(58.5)	210(53.4)	0.137	240(56.6)	242(55.8)	0.803
Family history,n(%)	18(5.6)	8(2.7)	0.069	17(5.0)	9(3.2)	0.283	14(4.6)	12(3.8)	0.616
Systolic blood pressure(mmHg)	120.09(105–132)	124.67(110–139)	**0.001**	120.65(106–132)	124.28(110–139)	**0.012**	120.71(106–132)	123.87(109–138)	**0.035**
Heart rate(bpm)	81.31(71–90)	81.48(70–92)	0.721	81.25(71–90)	81.56(70–91)	0.558	81.09(71–90)	81.68(70–91)	0.389
Shock index	0.70(0.57–0.79)	0.67(0.55–0.76)	**0.030**	0.69(0.57–0.78)	0.68(0.55–0.78)	0.147	0.69(0.56–0.78)	0.68(0.55–0.78)	0.500
Creatinine(umol/L)	80.37(62–86)	78.17(61–86)	0.430	79.37(62–86)	79.07(61–85)	0.371	79.80(62.5–86)	78.67(61–85)	0.378
LDL-C(mmol/L)	3.09(1.87–2.84)	2.49(1.95–2.97)	**0.019**	3.09(1.91–2.90)	2.46(1.91–2.94)	0.316	3.15(1.91–2.89)	2.45(1.91–2.94)	0.385
LVEF(%)	44.53(40–49)	46.47(42–50)	** < 0.001**	44.06(40–48)	47.15(44–50)	** < 0.001**	43.59(39–48)	47.34(45–50)	** < 0.001**
LVDd(cm)	5.54(5.26–5.72)	5.18(3.7–4.1)	** < 0.001**	5.53(5.25–5.70)	5.16(4.90–5.40)	** < 0.001**	5.55(5.3–5.74)	5.17(4.91–5.4)	** < 0.001**
IVSth(cm)	0.83(0.75–0.9)	0.97(0.85–1.05)	** < 0.001**	0.81(0.75–0.86)	1.00(0.90–1.05)	** < 0.001**	0.80(0.75–0.85)	1.0(0.90–1.05)	** < 0.001**
PWth(cm)	0.81(0.8–0.85)	0.95(0.89–1)	** < 0.001**	0.82(0.80–0.85)	0.95(0.85–1.0)	** < 0.001**	0.83(0.80–0.88)	0.92(0.82–1.0)	** < 0.001**
LA(cm)	4.09(3.75–4.2)	3.95(3.7–4.1)	**0.020**	4.07(3.75–4.2)	3.96(3.75–4.14)	0.237	4.09(3.77–4.2)	3.96(3.74–4.15)	**0.048**
Anterior wall,n(%)	216(51.9)	188(42.5)	**0.006**	242(52.0)	162(41.2)	**0.002**	250(59.0)	154(35.5)	** < 0.001**
Multivessel lesions,n(%)	164(37.1)	140(33.7)	0.291	166(35.7)	138(35.1)	0.858	146(34.4)	158(36.4)	0.546

The continous variables were presented as mean ± SD or median(IQR)

*LDL-C* Low density lipoprotein cholesterol, *LVEF* Left ventricular ejection fraction, *LVDd* Left ventricular diameter at the end of diastole, *IVSth* Intraventricular septal thickness, *PWth* Posterior wall thickness, *LA* Left atrium

### Survival analysis

**Fig. 2:**
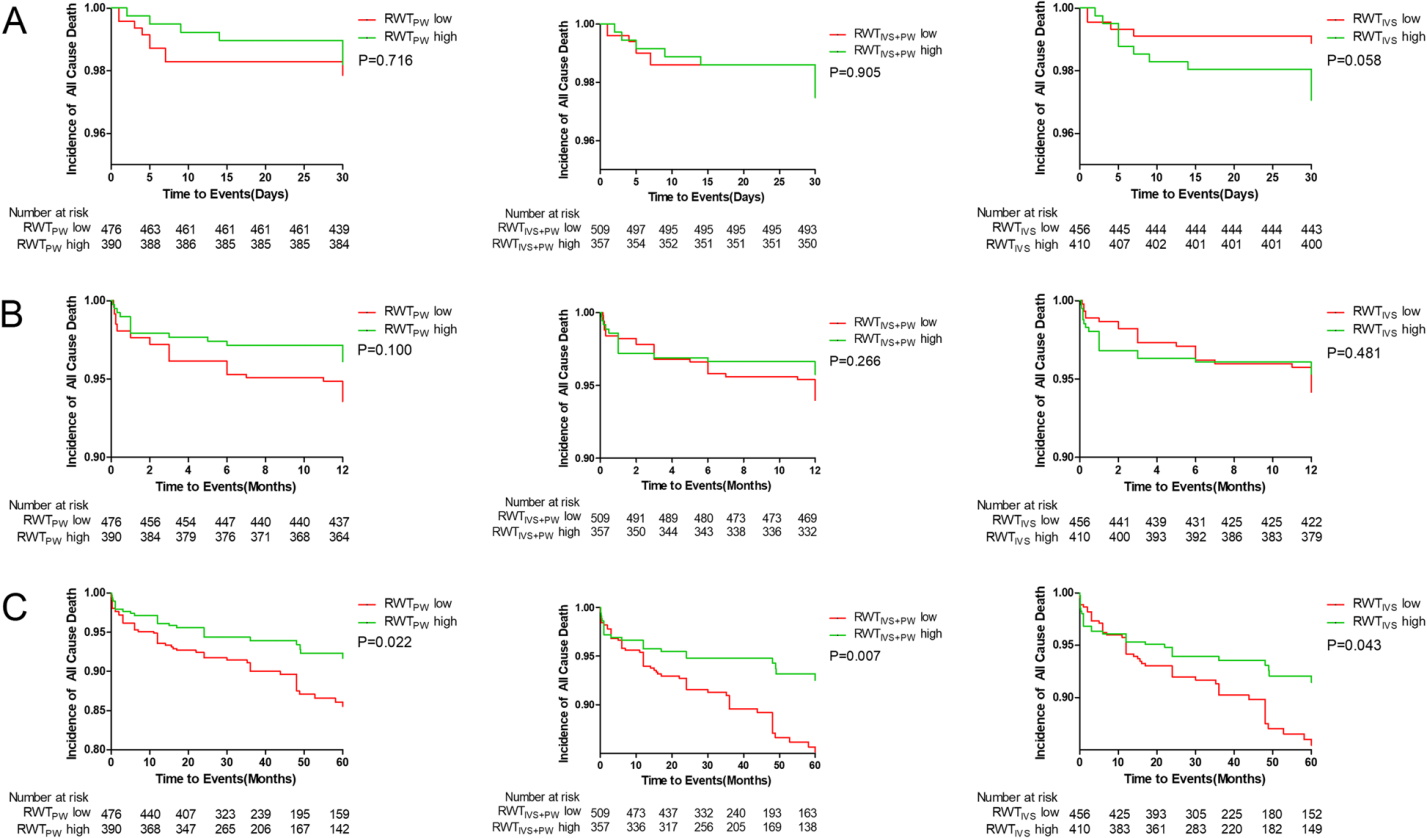
Survival analysis curve of all cause death at 30 days(**A**), 12 months(**B**) and 60 months(**C**)

**Fig. 3:**
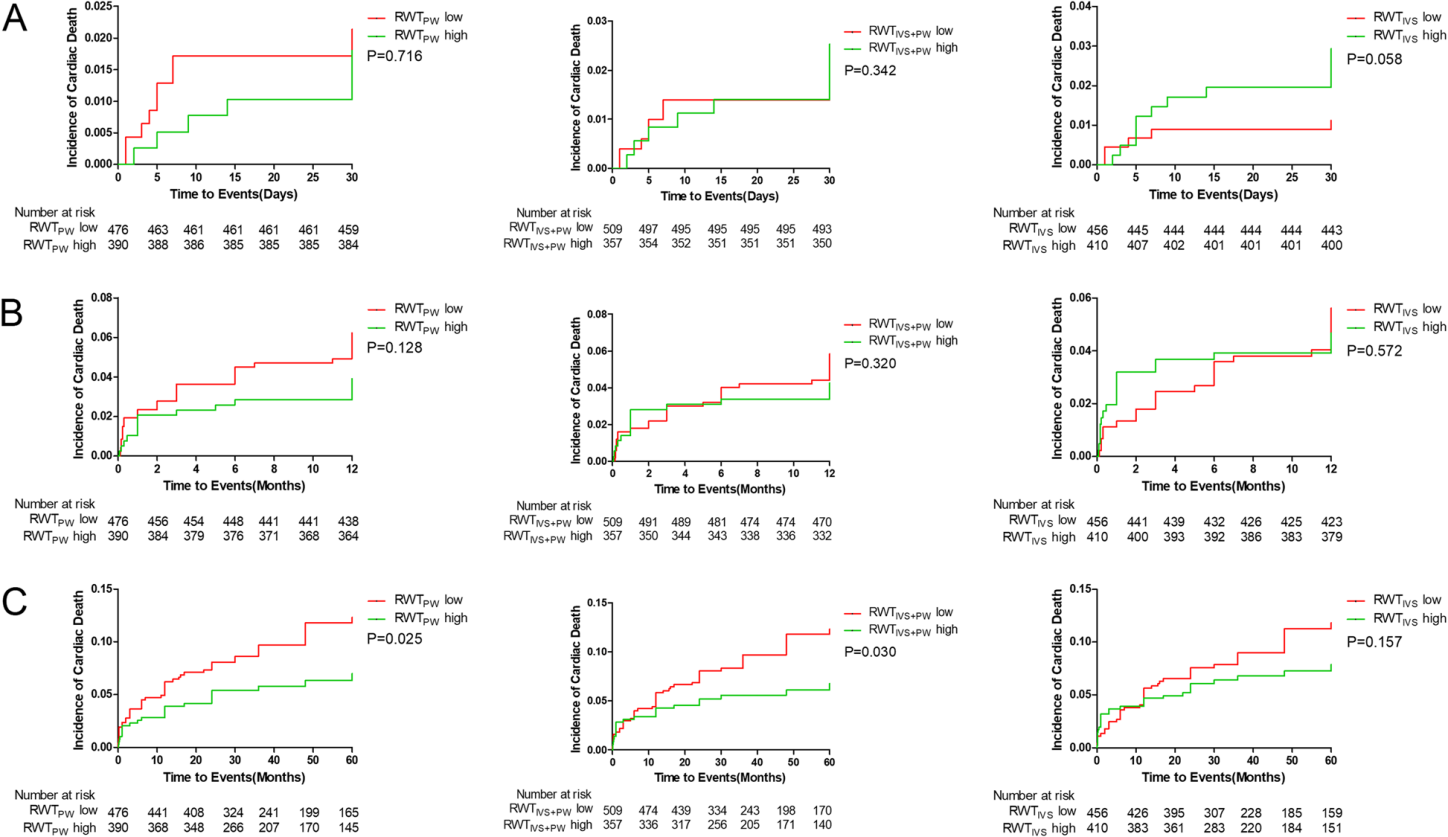
Survival analysis curve of cardiac death at 30 days(**A**), 12 months(**B**) and 60 months(**C**)

**Fig. 4:**
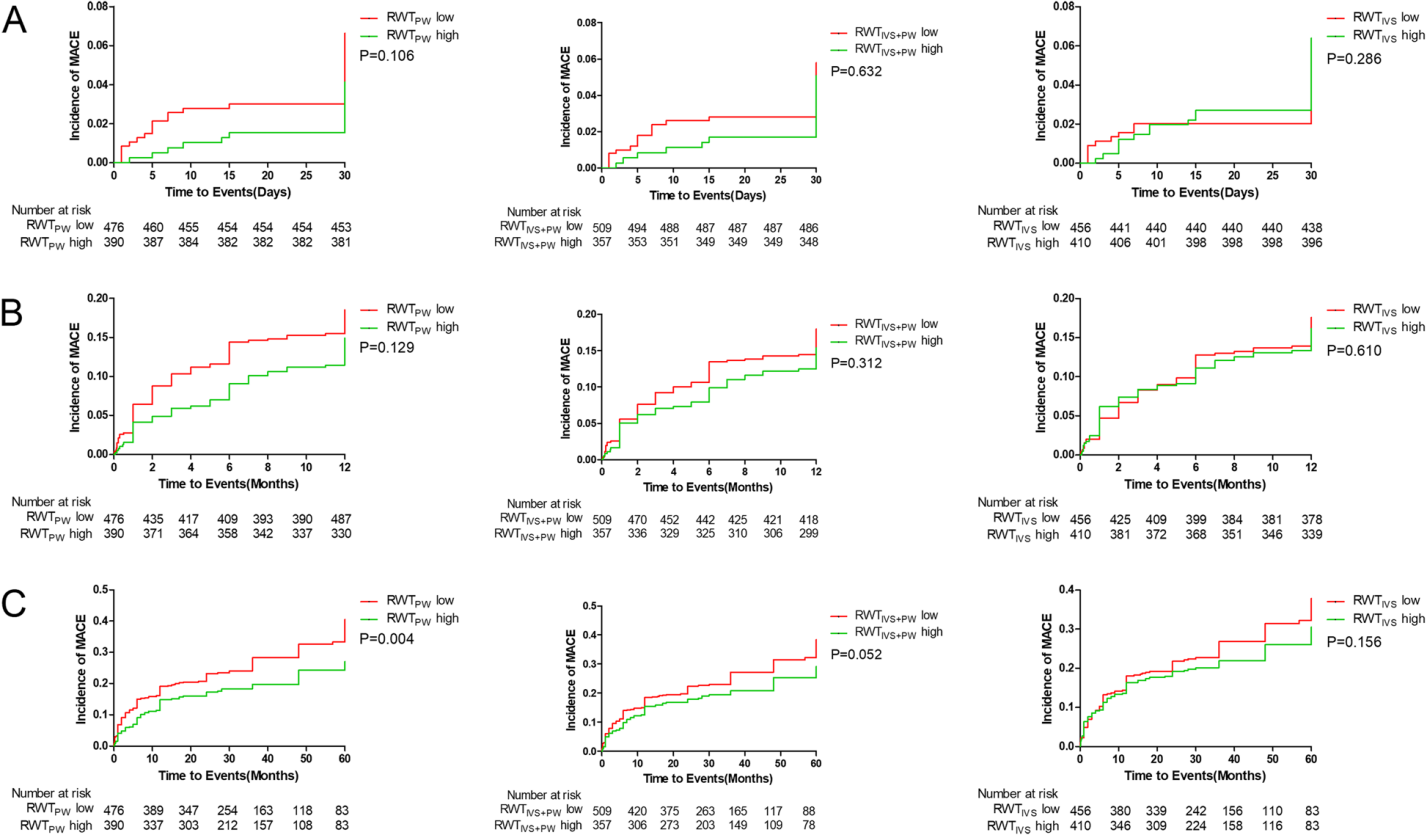
Survival analysis curve of MACE at 30 days(**A**), 12 months(**B**) and 60 months(**C**)

The median follow-up period was 54.3 months (22.0–78.7 months). During follow-up, 83(9.6%) patients died. Comparing low and high group of RWT_PW_, RWT_IVS+PW_ and RWT_IVS_, there was no significant difference in the incidence of all cause death, cardiac death and MACE at 30 days and 12 months (Fig. 2, 3 and 4). The incidence of the all cause death in 60 months was significantly higher in the low groups as compared to the high groups (RWT_PW_:11.5% vs 6.9%, *P* = 0.022; RWT_IVS+PW_:11.6% vs 6.4%, *P* = 0.007; RWT_IVS_:11.4% vs 7.3%, *P* = 0.043) (Fig. 2). The incidence of the cardiac death in 60 months was significantly different when RWT_PW_ and RWT_IVS+PW_ were used (RWT_PW_: 10.5% vs 6.2%, *P* = 0.025; RWT_IVS+PW_:10.2% vs 6.2%, *P* = 0.030) (Fig. 3). The incidence of MACE in 60 months was significantly different between the low groups and high group of RWT_PW_ (RWT_PW_:29.7% vs 21.3%, *P* = 0.004) (Fig. 4).

### Cox proportional hazard models for the endpoints

**Fig. 5:**
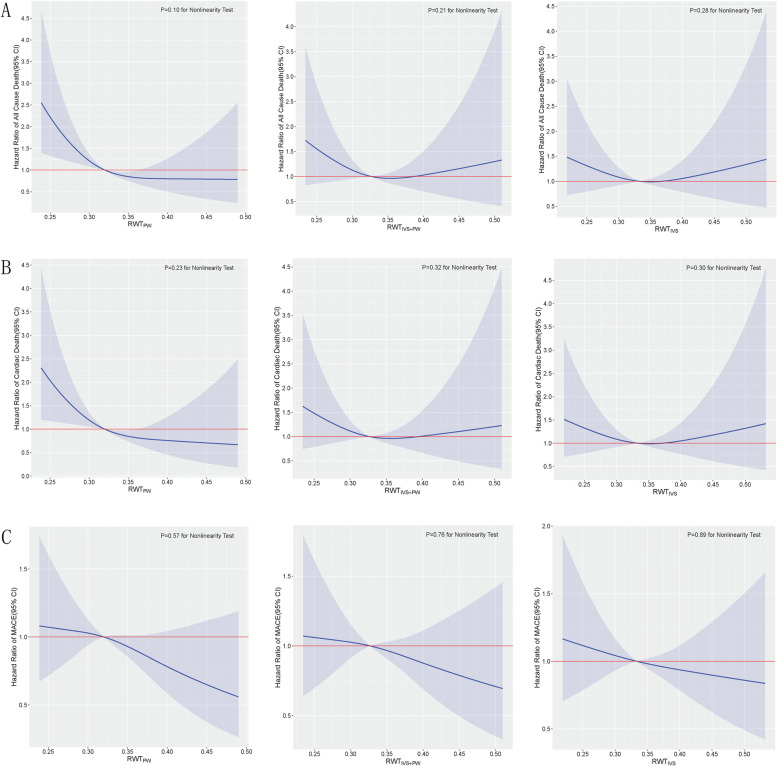
Multivariable adjusted hazrad ratios for all cause death(**A**), cardiac death(**B**) and MACE(**C**) according to three calculation methods of RWT on a continuous scale. Reference lines for no association are indicated by solid red lines at a hazard ratio of 1.0. Solid blue lines are multivariable adjusted hazrad ratios, with blue shadow showing 95% confidence intervals derived from restricted cubic spliners regression with three knots

The restricted cubic spline models were illustrated in Fig. 5, and no evidence of non-linearity was observed.

The crude and adjusted association of RWT_PW_, RWT_IVS+PW_ and RWT_IVS_ with all cause death, cardiac death, and MACE are presented in Table 2. Two models were used to adjust the covariates for evaluating the stability of the model. Model 1 included male sex, age, hypertension, diabetes, smoking, prior stroke and hyperlipidemia. Model 2 included sex, age, hypertension, diabetes, smoking, prior stroke, hyperlipidemia, creatinine, LVEF, shock index, Killip classification and multivessel lesions. Higher levels of RTW_PW_ were independently associated with a lower incidence of the cardiac death and MACE. The adjusted HR per 0.1 increase of RTW_PW_ for cardiac death and MACE were 0.38 and 0.68, respectively.

**Table 2:** Multivariate Cox regression analysis of the overall cohort

	RWT_PW_ (per 0.1 increased)			RWT_IVS+PW_ (per 0.1 increased)			RWT_IVS_ (per 0.1 increased)		
	HR	95%CI	*P* Value	HR	95%CI	*P* Value	HR	95%CI	*P* Value
All cause death									
Unadjusted	0.38	0.23–0.65	** < 0.001**	0.37	0.28–0.61	** < 0.001**	0.46	0.31–0.69	** < 0.001**
Adjusted with Model1	0.47	0.29–0.78	**0.003**	0.46	0.29–0.74	**0.001**	0.56	0.38–0.81	**0.002**
Adjusted with Model2	0.62	0.38–1.02	0.057	0.70	0.44–1.11	0.130	0.8	0.55–1.17	0.260
Cardiac death									
Unadjusted	0.38	0.22–0.66	**0.001**	0.36	0.21–0.61	** < 0.001**	0.45	0.29–0.68	** < 0.001**
Adjusted with Model1	0.47	0.28–0.80	**0.005**	0.45	0.27–0.74	**0.002**	0.54	0.36–0.81	**0.003**
Adjusted with Model2	0.55	0.33–0.89	**0.016**	0.70	0.44–1.11	0.130	0.81	0.54–1.21	0.310
MACE									
Unadjusted	0.68	0.53–0.91	**0.008**	0.68	0.53–0.89	**0.004**	0.73	0.59–0.91	**0.005**
Adjusted with Model1	0.73	0.56–0.95	**0.027**	0.74	0.57–0.95	**0.019**	0.78	0.64–0.97	**0.024**
Adjusted with Model2	0.74	0.57–0.98	**0.030**	0.82	0.63–1.06	0.130	0.86	0.69–1.07	0.180

Model 1: sex, age, hypertension, diabetes, smoking, prior stroke, hyperlipidemia

Model 2: sex, age, hypertension, diabetes, smoking, prior stroke, hyperlipidemia, creatinine, left ventricular ejection fraction (LVEF), shock index, killips classification, multivessel lesion

The patients were divided into anterior wall infarction subgroup and non-anterior wall infarction subgroup according to whether the anterior wall was involved. In the anterior wall subgroup, RWT_PW_, RWT_IVS+PW_ and RWT_IVS_ were all inversely associated with the incidence of the all cause death and cardiac death before adjustment. After adjusted by model 1 and model 2, there was no significant difference in the incidence of the all cause death, cardiac death and MACE (Table 3).

**Table 3:** Multivariate Cox regression analysis for the anterior infarction group

	RWT_PW_ (per 0.1 increased)			RWT_IVS+PW_ (per 0.1 increased)			RWT_IVS_ (per 0.1 increased)		
	HR	95%CI	*P* Value	HR	95%CI	*P* Value	HR	95%CI	*P* Value
All cause death									
Unadjusted	0.46	0.23–0.92	**0.029**	0.41	0.21–0.81	**0.010**	0.46	0.26–0.82	**0.008**
Adjusted with Model1	0.68	0.35–1.32	0.260	0.57	0.31–1.08	0.080	0.63	0.37–1.08	0.090
Adjusted with Model2	0.94	0.47–1.88	0.860	0.86	0.45–1.63	0.650	0.82	0.48–1.40	0.470
Cardiac death									
Unadjusted	0.49	0.24–1.00	**0.050**	0.44	0.22–0.88	**0.020**	0.49	0.30–0.87	**0.020**
Adjusted with Model1	0.64	0.33–1.24	0.180	0.61	0.32–1.16	0.130	0.66	0.38–1.14	0.140
Adjusted with Model2	1.07	0.56–2.07	0.830	0.79	0.41–1.52	0.470	0.80	0.46–1.39	0.420
MACE									
Unadjusted	0.89	0.63–1.27	0.510	0.83	0.60–1.17	0.300	0.84	0.63–1.11	0.230
Adjusted with Model1	0.96	0.66–1.38	0.870	0.99	0.71–1.38	0.970	0.97	0.73–1.30	0.860
Adjusted with Model2	0.97	0.68–1.38	0.850	1.01	0.72–1.41	0.970	0.99	0.73–1.32	0.930

Model 1: sex, age, hypertension, diabetes, smoking, prior stroke, hyperlipidemia

Model 2: sex, age, hypertension, diabetes, smoking, prior stroke, hyperlipidemia, creatinine, left ventricular ejection fraction (LVEF), shock index, Killips classification, multivessel lesion

In non-anterior wall subgroup, all three calculations of RWT were significantly associated with the incidence of the all cause death, cardiac death and MACE before adjusted. After adjusted by model 1 and model 2, only RWT_PW_ (HR:0.30, 95%CI:0.12–0.75, *P* = 0.010) was inversely associated with the all-cause death and cardiac death, while RWT_PW_ (HR:0.55, 95%CI:0.36–0.84, *P* = 0.006) and RWT_IVS+PW_ (HR:0.61, 95%CI:0.41–0.91, *P* = 0.014) were inversely associated with incidence of MACE (Table 4).

**Table 4:** Multivariate Cox regression analysis of the non-anterior infarction group

	RWT_PW_ (per 0.1 increased)			RWT_IVS+PW_ (per 0.1 increased)			RWT_IVS_ (per 0.1 increased)		
	HR	95%CI	*P* Value	HR	95%CI	*P* Value	HR	95%CI	*P* Value
All cause death									
Unadjusted	0.29	0.13–0.65	**0.002**	0.34	0.16–0.71	**0.004**	0.47	0.26–0.86	**0.015**
Adjusted with Model1	0.35	0.16–0.76	**0.009**	0.41	0.20–0.83	**0.014**	0.53	0.30–0.93	**0.034**
Adjusted with Model2	0.45	0.21–0.97	**0.042**	0.56	0.27–1.15	0.120	0.71	0.39–1.28	0.250
Cardiac death									
Unadjusted	0.26	0.11–0.62	**0.003**	0.30	0.13–0.69	**0.004**	0.44	0.23–0.85	**0.014**
Adjusted with Model1	0.27	0.11–0.67	**0.004**	0.32	0.14–0.73	**0.007**	0.48	0.25–0.93	**0.028**
Adjusted with Model2	0.30	0.12–0.75	**0.010**	0.44	0.19–1.02	0.054	0.54	0.19–1.02	0.054
MACE									
Unadjusted	0.50	0.32–0.78	**0.003**	0.56	0.37–0.84	**0.005**	0.68	0.48–0.96	**0.027**
Adjusted with Model1	0.52	0.34–0.81	**0.003**	0.58	0.39–0.86	**0.007**	0.69	0.49–0.96	**0.029**
Adjusted with Model2	0.55	0.36–0.84	**0.006**	0.61	0.41–0.91	**0.014**	0.72	0.51–1.01	0.052

Model 1: sex, age, hypertension, diabetes, smoking, prior stroke, hyperlipidemia

Model 2: sex, age, hypertension, diabetes, smoking, prior stroke, hyperlipidemia, creatinine, left ventricular ejection fraction (LVEF), shock index, Killips classification, multivessel lesion

**Fig. 6:**
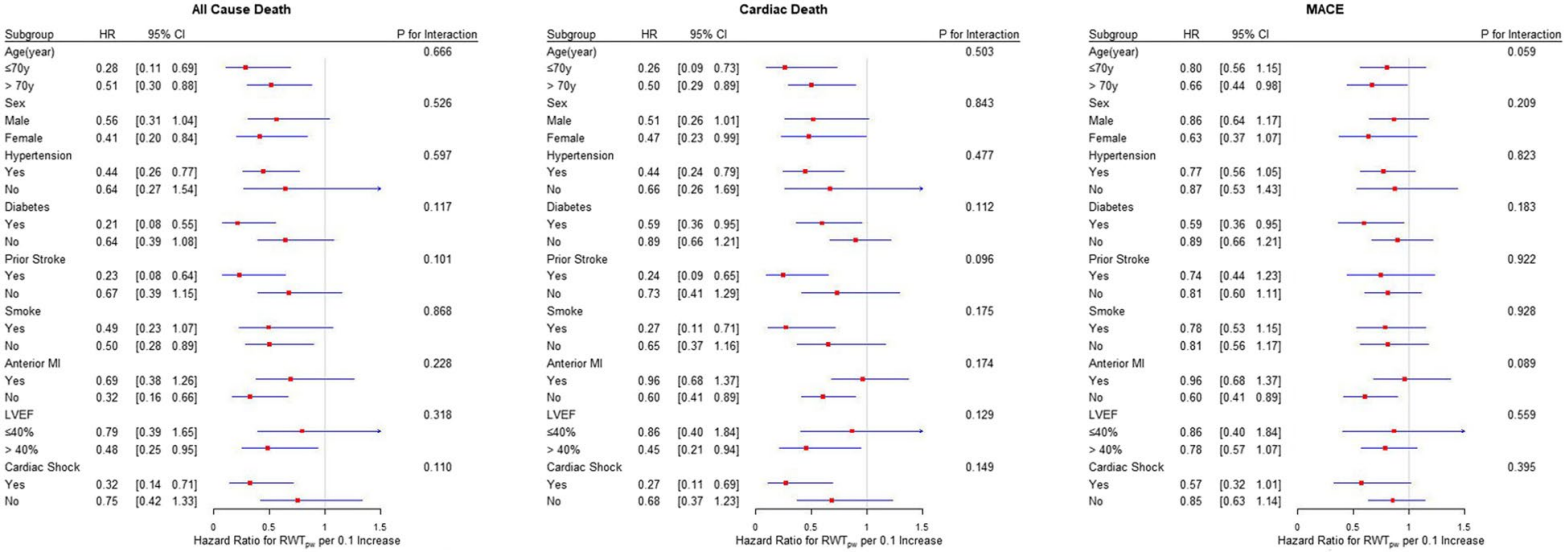
Subgroups analysis of all cause death, cardiac death and MACE for RWT_PW_

The interaction analysis of RWT_PW_ was analysed by age, gender, hypertension, diabetes, stroke history, smoking, anterior myocardial infraction, LVEF and cardiogenic shock. The endpoint events were all cause death, cardiac death and MACE in 60 months. Consequently, there were no interaction of RWT_PW_ with the above variates (Fig. 6).

## Discussion

The present study shows that RWT_PW_ is an independent and effective predictor of long-term cardiac death and MACE in patients with STEMI after pPCI. The patients with STEMI usually face substantial risks of long-term MACE although the cardiovascular events mainly occur within 1 months after pPCI [5]. Moreover, the occurrence of STEMI has been more prevalent during young people in recent years [2, 14]. Therefore, it is worthy to find the indicators which can predict the prognosis of patients with STEMI.

The rationale for investigating RWT as a prognosis marker is that RWT can reflect ventricular remodeling to a certain degree. When STEMI occurs, the heart does not change homogeneously, but changes according to the myocardium involved by the infarct-related vessels. The Framingham Heart Study firstly assessed the relationship between left ventricular geometry and clinical outcomes and demonstrated that patients with concentric hypertrophy had a poorest prognosis, followed by eccentric hypertrophy, concentric remodeling and normal morphology [15]. Yitschak Biton, et al. had described the relationship between the remodeling morphologies and the risk of ventricular tachyarrhythmia (VA) in patients with mild heart failure and RWT was found to be inversely associated with the risk of VA in patients with eccentric hypertrophy. Li L, et al. showed that RWT was an independent predictor of left ventricular systolic and diastolic dysfunctions in essential hypertension [16].

There are three methods to calculating RWT and the American Society of Echocardiography recommends RWT_PW_ for calculating RWT in clinical practice but several studies have found RWT_IVS+PW_ also had clinical significance [17, 18]. In the current study, we revealed that only RWT_PW_ had predictive value for all-cause death, cardiac death and MACE at 60 months in patients with non-anterior STEMI. None of the three calculations of RWT had the predictive value in the anterior STEMI cohort. This may be attributed to the fact that none of the three methods involved the index of the anterior wall and IVSD is only intraventricular septal thickness, which cannot fully reflect the degree of myocardial remodeling after anterior myocardial infarction. Survival analysis demonstrated that patients with lower RWT_PW_ or RWT_IVS+PW_ had significantly higher incidence of the all-cause death, cardiac death and MACE at 60 months as compared to those with higher RWT_PW_ or RWT_IVS+PW_. However, no significance was observed at 30 days and 12 months. This indicates that RWT has predictive value for long-term rather than short-term outcomes. Structural changes such as ventricular remodeling after myocardial infarction usually lasts for a long period and the adverse events accumulate as the time goes by. This could probably explain why RWT is a long-term independent predictor rather than short-term predictor.

The magnitude of RWT might mirror the extent of LV fibrosis and remodeling. The lower RWT is related to the thinner LV wall, the larger cardiac cavity, and the more severe necrosis of the involved myocardium. Cardiac remodeling can induce fibrosis, scar formation and subsequently lead to apoptosis of healthy cardiomyocytes, increased cardiac stiffness, decreased cardiac function and increased incidence of malignant arrhythmia [19]. Thus, the long-term clinical prognosis of patients with lower RWT value could be much worse.

## Limitations

The present study had several limitations. (1) The sample size was a bit small and the results may be biased to some degree. (2) It was an observational study, which had the intrinsic shortcomings. The biases were unable to be avoided completely despite of the adjustment of confounding factors using regression models. (3) There were many factors affecting the prognosis of STEMI patients, which need to be comprehensively evaluated.

## Conclusion

RWT_PW_, RWT_IVS+PW_ and RWT_IVS_ had no predictive value for the long-term clinical outcomes of patients with anterior myocardial infarction. On the contrary, RWT_PW_ had predictive value for long-term all cause death, cardiac death and MACE in patients with non-anterior myocardial infarction, which suggested that RWT_PW_, rather than RWT_IVS+PW_ or RWT_IVS_, was a reliable independent predictor.

## Data Availability

The datasets used and analysed during the current study available from the corresponding author on reasonable request.
